# CT texture analysis of pulmonary lesions in patients suspected for lung cancer

**DOI:** 10.1186/1470-7330-14-S1-S6

**Published:** 2014-10-09

**Authors:** MB Andersen, SW Harders, B Ganeshan, J Thygesen, HH Madsen, F Rasmussen

**Affiliations:** 1 Department of Radiology, Aarhus University Hospital, Aarhus, Denmark

## Objective

In this preliminary report we evaluate the impact of CT texture analysis (CTTA) of pulmonary lesions when compared to final tumour stage in patients with suspected lung cancer on contrast enhanced CT.

## Methods

Using texture analysis, we analysed 104 lesions in 104 patients suspected for lung cancer with a positive CT correlate. The analysis was performed using TexRAD (developed by TexRAD Ltd. UK). Histology was our reference standard. Malignancy was present in 92 lesions. CTTA comprised a filtration-histogram technique where filtration extracted and enhanced features of different sizes (fine, medium, coarse – scales) followed by histogram analysis using mean (M), entropy (E), uniformity (U), total number of voxels and kurtosis (K) within the entire volume of the suspected lesion. The operator performing the CTTA was blinded to the histological results.

## Results

In 58 malignant lesions with histologically verified TNM tumour stage, a Spearman’s rank correlation found significant positive correlations between Kurtosis and tumour stage at coarse filter scales. (ρ(58)=0.476, p<0.0005). We also found a significant positive correlation between total number of voxels and tumour stage on unfiltered data (ρ(58)=0.387, p=0.003)

## Conclusion

A significant correlation between texture figures and final tumour stage was found in patients with lung lesions suspected for lung cancer. Texture analysis may add complementary information to CE-CT.

